# Supplementary material for: Identification of photoperiod-induced specific miRNAs in the adrenal glands of Sunite sheep (Ovis aries)
Source: Front Vet Sci. 2022 Jul 22;9:888207. doi: 10.3389/fvets.2022.888207 (PMC9354845; doi:10.3389/fvets.2022.888207)
Supplement: Supplementary file 1 [file Data_Sheet_1.ZIP › Supplementary file/miRDeep2 file.zip!/miRDeep2 file/Know_Structure/SP42A2/oar-mir-493.pdf]

miRBase precursor : oar-mir-493
Total read count : 16022
oar-miR-493-5p read count : 11739
oar-miR-493-3p read count : 4281
remaining reads : 2

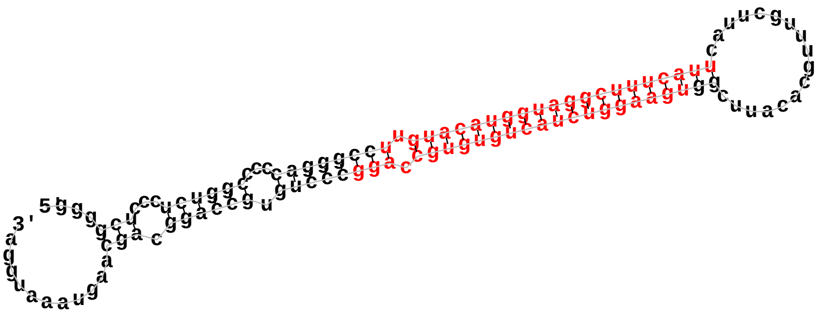

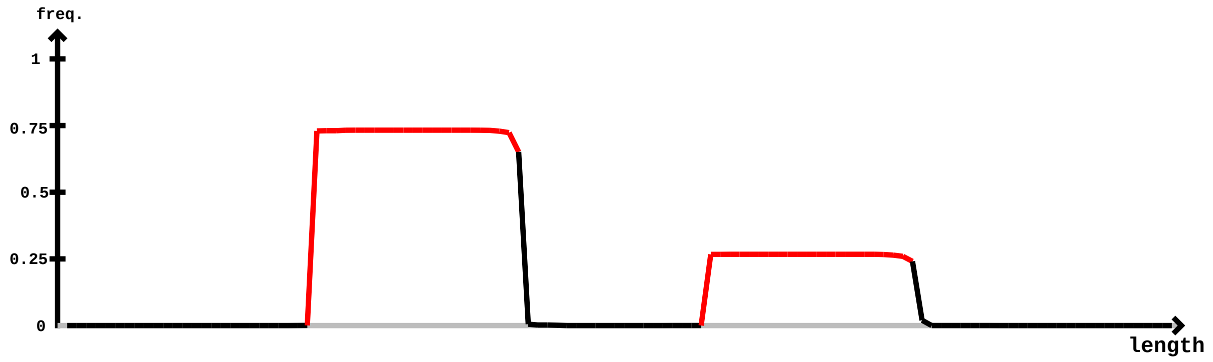


```
                          oar-miR-493-5p
                                                   oar-miR-493-3p
5'- ggggcucccucuggcccccagggccuuguacaugguaggcuuucauucauucguuugcacauucggugaaggucuacugugugccaggcccugugccaggcagcaaguaaaugga   -3'    exp
    ...(((...((((((...((((((((.((((((((((((((((((((.................)))))))))))))))))))).)))))))).)))))).))).........    reads    mm    sample
    .......................ccuuguacaugguaggcuuuc.......................................................................    2        0     seq
    .......................ccuuguacaugguaggcuuuca......................................................................    1        0     seq
    ........................cuuguacaugguaggcuuucauu....................................................................    5        0     seq
    .........................uuguacaugguaggcuu.........................................................................    3        0     seq
    .........................uuguacaugguaggcuuu........................................................................    9        0     seq
    .........................uuguacaugguaggcuuuc.......................................................................    46       0     seq
    .........................uugCacaugguaggcuuuc.......................................................................    1        1     seq
    .........................uuguacaugguaggcuuuca......................................................................    85       0     seq
    .........................uugCacaugguaggcuuuca......................................................................    1        1     seq
    .........................uuguacaugguaggcuuucau.....................................................................    1154     0     seq
    .........................uuguacaugguaggcuuucauu....................................................................    10337    0     seq
    .........................uuguacaugguaggcuuucauuc...................................................................    48       0     seq
    .........................uuguacaugguaggcuuucauucau.................................................................    1        0     seq
    ..........................uguacaugguaggcuuucau.....................................................................    1        0     seq
    ..........................uguacaugguaggcuuucauu....................................................................    9        0     seq
    ..........................uguacaugguaggcuuucauuc...................................................................    2        0     seq
    ............................uacaugguaggcuuucauu....................................................................    2        0     seq
    ............................uacaugguaggcuuucauucau.................................................................    11       0     seq
    ............................uacaugguaggcuuucauucauu................................................................    18       0     seq
    .............................acaugguaggcuuucauucau.................................................................    1        0     seq
    .............................acaugguaggcuuucauucauu................................................................    2        0     seq
    ..............................................................ucggugaaggucuacugugugc...............................    1        0     seq
    .................................................................gugaaggucuacugugugccag............................    1        0     seq
    .................................................................gugaaggucuacugugugccagg...........................    1        0     seq
    ..................................................................ugaaggucuacugugugU...............................    5        1     seq
    ..................................................................ugaaggucuacugugugc...............................    8        0     seq
    ..................................................................ugaaggucuacugugugG...............................    1        1     seq
    ..................................................................ugaaggucuacugugugcc..............................    37       0     seq
    ..................................................................ugaaggucuacugugugcca.............................    71       0     seq
    ..................................................................ugaagguUuacugugugccag............................    1        1     seq
    ..................................................................ugaaggucuGcugugugccag............................    1        1     seq
    ..................................................................ugaaggCcuacugugugccag............................    2        1     seq
    ..................................................................ugaaggucuacugugugccag............................    297      0     seq
    ..................................................................ugaagAucuacugugugccagg...........................    3        1     seq
```

```
                         oar-miR-493-5p
                                                                  oar-miR-493-3p
ggggcucccucuggcccccagggccuuguacaugguaggcuuucauucauucguuugcacauucggugaaggucuacugugugccaggcccugugccaggcagcaaguaaaugga

..................................................................ugaagguUuacugugugccagg...........................    2       1       seq
..................................................................ugaaggucuacugugugccagg...........................    3499    0       seq
..................................................................ugaaggucuacuAugugccagg...........................    8       1       seq
..................................................................ugaaUgucuacugugugccagg...........................    4       1       seq
..................................................................ugaaggCcuacugugugccagg...........................    9       1       seq
..................................................................ugaaggucuGcugugugccagg...........................    12      1       seq
..................................................................ugaaggucuacuguguUccagg...........................    2       1       seq
..................................................................ugaaggucuGcugugugccaggc..........................    2       1       seq
..................................................................ugaaggCcuacugugugccaggc..........................    1       1       seq
..................................................................ugaaggucuacugugugccaggc..........................    309     0       seq
....................................................................aaggucuacugugugccagg...........................    1       0       seq
....................................................................aaggucuacugugugccaggc..........................    4       0       seq
..........................................................................uacugugugccaggcccugugc...................    1       0       seq
```
